# LFA‐1‐Engineered Extracellular Vesicles Deliver CD59 to Protect Endothelial Integrity in Acute Lung Injury

**DOI:** 10.1002/advs.78673

**Published:** 2026-11-10

**Authors:** Yin Zhu, Shravani Etrouth, Payaningal R. Somanath, S. Priya Narayanan, Yutao Liu, Xiaoyun Wang, Duo Zhang

**Affiliations:** ^1^ Clinical and Experimental Therapeutics Department of Clinical and Administrative Pharmacy College of Pharmacy University of Georgia Augusta Georgia USA; ^2^ Augusta VA Healthcare System Augusta Georgia USA; ^3^ Department of Ophthalmology Mayo Clinic Rochester Minnesota USA; ^4^ Pulmonary, Critical Care and Sleep Medicine Department of Medicine Baylor College of Medicine Houston Texas USA

**Keywords:** bacterial infection, complement system, endothelial cell, lung vascular leakage, lymphocyte function‐associated antigen‐1, membrane attack complex

## Abstract

Acute lung injury (ALI) is a life‐threatening disorder characterized by pulmonary endothelial injury, vascular leakage, and hypoxemia, yet no targeted therapies are available to protect the lung vasculature. Dysregulated complement activation contributes to endothelial dysfunction through excessive deposition of the membrane attack complex (MAC), which disrupts endothelial barrier integrity. CD59 is a membrane‐bound complement inhibitor that protects endothelial cells (ECs) from MAC‐mediated injury, but its role and therapeutic potential in ALI remain unclear. In this study, we found that endothelial CD59 was significantly reduced during bacteria‐induced ALI and that *Cd59* deficiency exacerbated pulmonary vascular leakage in mice. To supplement endothelial CD59, we engineered extracellular vesicles (EVs) expressing lymphocyte function‐associated antigen‐1 (LFA‐1) (EV^LFA−1^) to enhance delivery to activated endothelium. CD59‐loaded EVs (EV^LFA−1^‐CD59) preferentially targeted ECs, reduced endothelial MAC deposition and apoptosis, and preserved endothelial barrier integrity in experimental ALI. EV^LFA−1^‐CD59 also improved survival following bacterial infection. Collectively, these data establish diminished endothelial CD59 levels as a critical driver of complement‐driven vascular injury and highlight EV^LFA−1^‐mediated CD59 delivery as a promising therapeutic strategy for ALI.

## Graphical abstract

LFA‐1‐engineered extracellular vesicles preferentially target pulmonary endothelium and deliver CD59, thereby inhibiting membrane attack complex formation, preserving vascular integrity, and attenuating acute lung injury. This strategy reveals a targeted approach for protecting the pulmonary vasculature from complement‐mediated damage.

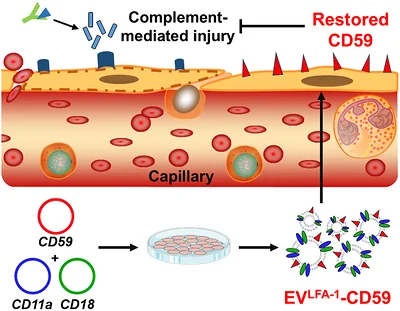

## 1 Introduction

Acute lung injury (ALI) and acute respiratory distress syndrome (ARDS) remain major causes of morbidity and mortality in critically ill patients, with death rates persistently ranging from 30%–40% despite improvements in critical care management [1, 2, 3, 4, 5]. The acute phase of ALI is characterized by pulmonary capillary leak, alveolar edema, and surfactant dysfunction, leading to diffuse alveolar collapse and impaired gas exchange [6]. ALI can result from multiple underlying causes, such as sepsis, pneumonia, or trauma, contributing to clinical heterogeneity and diagnostic challenges [3]. Both experimental and clinical studies indicate that endothelial cell death is a central mechanism contributing to vascular barrier disruption and disease progression [7, 8, 9]. However, no effective pharmacological therapy has demonstrated clinical efficacy [10, 11]. These challenges highlight an unmet need to define the molecular pathways governing endothelial injury and to develop innovative interventions that attenuate inflammation and vascular leakage, thereby improving survival in ARDS patients.

The complement system is a critical component of innate immunity and plays a key role in the pathogenesis of ALI [12]. Dysregulated complement activation promotes pulmonary endothelial dysfunction, inflammation, and cell death, which together precipitate vascular leakage, alveolar flooding, and impaired gas exchange [12, 13]. Mechanistically, the terminal complement complex C5b‐9 (membrane attack complex, MAC) and its soluble form sC5b‐9 have been shown to disrupt pulmonary microvascular endothelial integrity by activating RhoA and promoting myosin light chain phosphorylation, leading to cytoskeletal contraction and junctional disassembly [14]. CD59 is a cell surface glycoprotein encoded by the human *CD59* gene. It is highly expressed on vascular ECs [15]. The *CD59* gene is duplicated in mice, with two copies termed *Cd59* (also known as *Cd59a* or protectin) and *Cd59b*. *Cd59* is the primary regulator of complement activation and MAC deposition in mice [16, 17]. CD59 inhibits MAC deposition by binding complement components C8 and/or C9 during assembly, thereby preventing the incorporation of multiple C9 molecules and protecting cells from complement‐mediated lysis [18, 19]. Despite its established role in complement regulation, the contribution of CD59 to pulmonary endothelial barrier integrity in ALI remains unclear. Understanding how complement‐endothelial interactions regulate cell death and vascular integrity will be essential for developing novel therapeutic strategies.

Extracellular vesicles (EVs) are membrane‐delimited vesicles released by most cells that transport proteins, lipids, and nucleic acids to recipient cells, thereby regulating intercellular communication and tissue homeostasis [20]. Growing evidence indicates that EV cargo is associated with reduced inflammation, preserved barrier integrity, and improved outcomes in ALI models. Our previous study demonstrated that EV‐encapsulated Club Cell Protein 16 protects mice from lipopolysaccharide (LPS)‐ or bacteria‐induced ALI through its anti‐inflammatory and DNA damage repair effects [21]. Endothelial progenitor cell EVs that transfer microRNA‐126 reduce LPS‐induced ALI, improve endothelial junctions and survival, and illustrate nucleic‐acid cargo‐mediated vascular protection [22]. Mesenchymal stromal cell‐derived EVs transport *angiopoietin‐1* mRNA to the lung, leading to endothelial stabilization and reduced edema [23, 24]. In addition, Clayton et al. reported that EVs derived from antigen‐presenting cells carry membrane‐associated complement regulatory proteins, including CD46, CD55, and CD59, which protect EVs from complement‐mediated lysis [25]. Thus, EV platforms may serve as vehicles for targeted delivery of complement regulatory cargo to endothelial cells (ECs), thereby attenuating complement‐mediated endothelial injury and vascular leakage in ALI.

In this study, we found that CD59 was significantly decreased in lung ECs following *Klebsiella pneumoniae* (*K. p*) infection. Functionally, *Cd59* knockout (*Cd59* ^−/−^) mice exhibited worsened outcomes compared with wild‐type (WT) controls, including reduced survival, increased pulmonary edema, exacerbated tissue injury, and heightened vascular leakage. Consistent with this finding, endothelial MAC deposition and cleaved Caspase‐3 levels were significantly elevated in *Cd59* ^−/−^ mice compared to WT controls, indicating increased endothelial apoptosis associated with *Cd59* deficiency.

Intravenous administration of EV^LFA‑1^ resulted in preferential targeting to endothelial cells in the lungs of mice with ALI. Building on this platform, we generated CD59‑loaded EV^LFA‑1^ (EV^LFA−1^‐CD59) and administered them intravenously following *K. p* infection. Treatment with EV^LFA‑1^‐CD59 markedly reduced pulmonary edema, vascular leakage, MAC deposition, and endothelial apoptosis. Collectively, these findings demonstrate, for the first time, that loss of endothelial CD59 is a key driver of complement‑mediated vascular injury in ALI, and provide compelling proof‑of‑concept that engineered EV‑mediated delivery of CD59 can restore endothelial barrier integrity and mitigate bacteria‑induced lung injury.

It is well established that Lymphocyte function‐associated antigen‑1 (LFA‑1) is a heterodimeric integrin expressed on leukocytes that binds intercellular adhesion molecule‐1 (ICAM‐1) to mediate firm adhesion to the endothelium, thereby supporting immune cell trafficking [26]. To restore endothelial CD59 levels during bacteria‐induced ALI, we employed LFA‐1engineered EVs (EV^LFA−1^), which exhibit enhanced uptake by ICAM‐1‐expressing ECs [27]. Intravenous administration of EV^LFA−1^ resulted in preferential targeting to lung ECs in a mouse model of ALI. Building on this platform, we generated EV^LFA−1^‐CD59 and administered it intravenously to mice following *K. p* infection. Treatment with EV^LFA−1^‐CD59 significantly reduced pulmonary edema, vascular leakage, MAC deposition, and endothelial apoptosis. Collectively, these findings demonstrate, for the first time, that loss of endothelial CD59 is a key driver of complement‑mediated vascular injury in ALI, and provide compelling proof‑of‑concept that engineered EV‐mediated delivery of CD59 can restore endothelial barrier integrity and mitigate bacteria‑induced lung injury.

## 2 Materials and Methods

### 2.1 Antibodies

Primary and secondary antibodies used in this study, including their applications, dilutions, manufacturers, and catalog numbers, are listed in Table S1.

### 2.2 Animal Studies

C57BL/6 (Strain #: 000664) or *Cd59* ^−^ *^/^* ^−^ (Strain #: 037301) mice were purchased from Jackson Laboratory. Male and female mice aged 8–10 weeks were utilized in an approximately 1:1 ratio for all experiments. To minimize bias, animals were randomly assigned to experimental groups. The murine model of ALI was established as described previously [21]. Mice were administered 50 µL of PBS or 50 µL of PBS containing *Klebsiella pneumoniae* (*K. p*, ATCC 43186) by intratracheal instillation (i.t.). Two bacterial inocula were used: 1 × 10^4^ CFU per mouse to induce acute lung injury, and 1 × 10^3^ CFU per mouse for survival studies. Mice were euthanized 24 h post‐infection (p.i.), and the lung tissues were harvested for further analysis. To assess pulmonary edema, the whole lung tissues were dissected immediately following euthanasia. The wet weight was measured at the time of tissue excision, after which the samples were dried in an oven at 70°C for 48–72 h and reweighed to obtain a constant dry weight. The lung wet‐to‐dry weight ratio was then calculated by dividing the wet weight by the corresponding dry weight. The bronchoalveolar lavage (BAL) was performed as previously described [28]. Briefly, following euthanasia, mouse lungs were lavaged with a total of 1.8 mL PBS (0.6 mL × 3). The concentration of red blood cells (RBCs) in BAL fluid (BALF) was quantified using a hemocytometer as a direct indicator of alveolar‐capillary barrier disruption. For histologic analysis, lung tissues were collected and processed for cryosectioning, and lung sections were subjected to hematoxylin and eosin (H&E) staining in the Histology Core Laboratory at Augusta University. The histologic lung injury score was assessed in a double‐blinded condition as previously described [29]. The measurement of lung vascular permeability in vivo with Evans Blue was performed as described [30]. In brief, mice were injected intravenously via the tail vein with 200 µL of 0.5% Evans Blue solution. After 1 h, mice were euthanized, and lungs were harvested and incubated in formamide at 55°C for 48 h to extract the dye. Evans Blue extravasation was quantified by measuring absorbance at 620 nm (OD_620_) using a microplate reader. For the survival test, WT and *Cd59* ^−^ *^/^* ^−^ mice (*n* = 28 per group) were infected with *K. p* (10^3^ CFU per mouse) via i.t. In a separate experiment, WT mice (*n* = 24 per group) received EV^LFA−1^ and EV^LFA−1^‐CD59 24 h after intratracheal infection with *K. p* (10^3^ CFU per mouse). Survival and body weight loss were monitored every 12 h for 7 days p.i. Kaplan–Meier survival plots were generated and analyzed using the Gehan–Breslow test. The H1N1 (A/California/04/2009) infection model was performed as described previously [31]. An LD_20_ inoculum (1000 PFU) was used to evaluate lung inflammation and injury readouts as compared with uninfected control (UC) WT mice, which received PBS. The LPS‐induced mouse model of ALI was established as previously described [21, 32]. Briefly, mice received an intratracheal instillation of 5 µg LPS (*Escherichia coli* O111:B4, Sigma–Aldrich) diluted in 50 µL PBS to induce ALI. All animal procedures were approved by the Institutional Animal Care and Use Committee of the Charlie Norwood Veterans Affairs Medical Center and were conducted in accordance with ARRIVE guidelines.

### 2.3 Cell Culture and Plasmid Transfection

HULEC‐5a (CRL‐3244) was purchased from American Type Culture Collection (ATCC) and cultured with EGM Endothelial Cell Growth Medium BulletKit (LONZA). Human embryonic kidney cells (HEK293T) were obtained from American Type Culture Collection and maintained in DMEM with 10% FBS. All cells were cultured at 37°C in a humidified atmosphere with 5% CO_2_‐in air. Human LFA‐1 alpha (CD11a, Addgene plasmid # 8630) and Integrin beta 2 (CD18, Addgene plasmid # 8640) expression plasmids were gifts from Timothy Springer [33]. The human CD59 expression plasmid (Addgene plasmid # 50378) was a gift from Reika Watanabe [34]. Plasmid pcDNA3.1 (Thermo Fisher Scientific) was used as an empty vector control. The plasmids of CD11a, CD18, and/or CD59 were co‐transfected at a 1:1:1 ratio into HEK293T cells with Lipofectamine 3000 according to the manufacturer's protocol (Thermo Fisher Scientific). In vitro *K. p* infection was performed as previously described [21]. The concentration of *K. p* was determined by measuring OD_600_ with a plate reader. A total of 1 × 10^6^ cells were incubated with 5 × 10^6^ colony‐forming units (CFU) of *K. p* (MOI = 5) for 1 h. The cells were washed with PBS three times and incubated with 50 µg/mL gentamicin to eliminate any remaining bacteria. HULEC‐5a cells were incubated with 10% heat‐inactivated serum or 10% complement‐preserved normal human serum (BIOIVT, gender pooled) in the presence of 100 µg EV^LFA−1^ or EV^LFA−1^‐CD59 for 24 h. The cells were fixed for future analysis.

### 2.4 Primary Murine Lung ECs Isolation

Isolation of primary murine lung ECs was performed as described [35]. Briefly, mouse lungs from WT or *Cd59* ^−^ *^/^* ^−^ mice were harvested and transferred into a culture dish containing 0.1% collagenase (Roche) in 1% Penicillin–Streptomycin (Thermo Fisher Scientific)‐RPMI 1640 (Gibco). The lung tissues were minced with sterilized scissors and chopped with autoclaved blades. The lung tissue in RPMI containing 0.1% Collagenase was incubated for 30 min at 37°C. Fresh RPMI medium was used to wash the 70 µm cell strainer, and cell suspensions were passed through the strainer and then centrifuged at 200 × g for 8 min at 4°C. The cell pellet was washed with fresh RPMI medium, centrifuged again, and resuspended in complete medium containing 80% DMEM/F‐12 (Gibco), 20% Heat‐inactivated FBS (Sigma), MesoEndo cell growth supplements (Sigma), onto 0.1% gelatin (Sigma)‐coated plate. Immunoselection was applied to the cells when small colonies of endothelial‐like cells were present by using dynabeads (Sheep anti‐Rat IgG, Thermo Fisher Scientific) with CD102 and CD31 antibodies. Cells were then cultured on 0.1% gelatin‐coated plate with complete medium and maintained for experimental use.

### 2.5 EV Purification and Characterization

EVs were isolated from conditioned medium of transfected HEK293T cells as previously described [21, 36]. Cells were cultured in DMEM containing 1% FBS, and conditioned medium was collected 48 h after transfection. For each preparation, conditioned medium was sequentially centrifuged at 500 × g for 5 min and 2000 × g for 20 min to remove cells and reduce cellular debris. Conditioned medium was then mixed with an equal volume of 2× polyethylene glycol (PEG) precipitation buffer (16% PEG and 1 M NaCl) and incubated overnight at 4°C with gentle rocking. Samples were then centrifuged at 3000 × g for 1 h to pellet the EV‐enriched fraction. The EV pellet was resuspended in sterile PBS and subjected to ultracentrifugation at 120 000 × g for 2 h at 4°C using an Optima MAX‐XP ultracentrifuge equipped with an MLA‐130 fixed‐angle rotor (Beckman Coulter). The purified EV pellet was resuspended in PBS for downstream experiments. Negative‐staining TEM and immunogold‐labeled TEM were performed as previously described [21]. Briefly, EVs were probed with an anti‐CD59 monoclonal antibody (MA5‐32588, Thermo Fisher Scientific) and images were acquired using a JEM‐1230 transmission electron microscope (JEOL USA) at the Electron Microscopy Core Laboratory (RRID: SCR_026810) of Augusta University. Nanoparticle tracking analysis (NTA) was conducted using the ZetaView PMX 110 (Particle Metrix) in Dr. Yutao Liu's lab at the Department of Cellular Biology and Anatomy of Augusta University. EV samples were diluted in sterile PBS before analysis, and particle concentration was quantified based on the recorded measurements [37].

### 2.6 Organ and Cellular Biodistribution of EVs

To track EV distribution after intravenous injection, EVs were labeled using the PKH26 Fluorescent Cell Linker Kit (Sigma) or with DiR near‐infrared (NIR) fluorescence dye (XenoLight, 125964), as previously reported [38]. WT mice (*n* = 4) were studied under untreated conditions or following administration of LPS (5 µg per mouse). 24 h after LPS administration, mice received a tail‐vein injection of fluorescently labeled control EVs (EV^Con^) or LFA‐1‐engineered EVs (EV^LFA−1^) at a dose of 200 µg per mouse, based on EV protein content. For organ biodistribution analysis, EVs were labeled with DiR near‐infrared fluorescent dye (XenoLight). Briefly, 100 µg of EV protein was suspended in 25 µL PBS (4 µg/µL) and mixed with an equal volume of DiR solution (25 µL, 640 µg/mL). The resulting 50 µL labeling mixture contained 2 µg/µL EV protein and 320 µg/mL DiR. The mixture was incubated for 30 min at room temperature. Following labeling, the preparations were passed through PBS‐equilibrated Zeba Spin Desalting Columns (Thermo Scientific) to remove unbound dye. DiR‐labeled EVs were injected via the tail vein, and mice were euthanized 2 h after injection. The brain, heart, lungs, liver, kidneys, and spleen were collected and imaged using a Spectral AMI X Bioluminescent/Fluorescent Imaging System (Spectral Instruments Imaging, a Bruker company). Fluorescence signals were quantified using Aura software. To assess pulmonary endothelial localization, EV^Con^ and EV^LFA−1^ were labeled with the PKH26 Red Fluorescent Cell Linker Kit (Sigma–Aldrich) according to the manufacturer's instructions. PKH26‐labeled EVs were administered by tail‐vein injection. Mice were euthanized 24 h after injection, and organs were collected, fixed in 4% paraformaldehyde for 24 h, and processed for frozen sectioning by the institutional core facility. Lung, liver, and spleen sections were stained with anti‐CD31 antibody (553369, BD Pharmingen, 1:200 dilution), followed by IgG‐Alexa Fluor 488 secondary antibodies (Thermo Fisher Scientific). Sections were mounted using DAPI‐containing mounting medium (ab104139, Abcam) to stain the nuclei. Imaging was conducted using a Carl Zeiss Observer Z1 microscope, and the fluorescence intensity was quantified using ImageJ software.

### 2.7 Western Blot Analysis

Western blot analyses were performed as previously described [39]. Murine lung tissues, cells, or EV samples were lysed using RIPA buffer (RPI Research Products) supplemented with protease inhibitor (Thermo Fisher Scientific). Total protein lysates were resolved by SDS‐PAGE and subsequently transferred onto PVDF membranes (Biorad). The PVDF membranes were blocked with bovine serum albumin (Sigma) and incubated with primary anti‐CD11a antibody, anti‐CD18 antibody, monoclonal anti‐CD9 antibody, monoclonal anti‐Flotillin 1 antibody, polyclonal anti‐SP‐1 antibody, and monoclonal anti‐CD59 antibody overnight. The membrane was then washed with TBST solution and incubated with a secondary anti‐rabbit HRP‐conjugated antibody for 1 h. To verify β‐actin expression, membranes were stripped using stripping buffer (Thermo Fisher Scientific) and re‐incubated overnight with a monoclonal anti‐β‐actin antibody (A5441, MilliporeSigma), followed by incubation with an HRP‐conjugated anti‐mouse secondary antibody. Bands were visualized using the ChemiDoc imaging system (BioRad), and the analysis was performed using ImageJ software.

### 2.8 ELISA

WT mice (*n* = 6) were infected with *K. p* (1 × 10^4^ CFU per mouse), and BALF was collected 24 h after infection. BALF concentrations of CD59a, C5a, and C9 were quantified using the Mouse CD59a/MAC‐IP ELISA Kit (RayBiotech), Mouse C5a ELISA Kit (RayBiotech), and Mouse Complement Component 9 ELISA Kit (MyBioSource), according to the manufacturers’ protocols. For CD59a and C5a assays, standards and BALF samples were added to antibody‐precoated microplates and incubated overnight at 4°C. Following washing, biotinylated detection antibodies were added and incubated for 1 h at room temperature, followed by streptavidin–horseradish peroxidase for 45 min at room temperature, with washing between steps. Color development was performed using tetramethylbenzidine substrate for 30 min at room temperature, and the reaction was terminated by adding stop solution. For the C9 assay, BALF samples were combined with biotinylated anti‐C9 antibody and streptavidin–horseradish peroxidase and incubated for 60 min at 37°C. Standard wells were prepared according to the kit instructions. Plates were washed five times, followed by the addition of TMB substrate solutions and incubation for 10 min at 37°C in the dark. The reaction was terminated by adding stop solution to each well. Absorbance was measured at 450 nm using a microplate reader, and analyte concentrations were calculated from the corresponding standard curves generated for each assay.

### 2.9 RNA Preparation, Reverse Transcription, and RT‐qPCR

Total RNA was extracted using RNeasy Plus Kits (Qiagen) following the manufacturer's instructions. cDNA was synthesized using the high‐capacity cDNA reverse transcription kit (Thermo Fisher Scientific) following the manufacturer's instructions. Human and mouse *TBP* were used as housekeeping genes. Primers used in the study were purchased from Integrated DNA Technologies (Coralville, IA, USA). PowerUP Green Master Mix (Thermo Fisher Scientific) was used for RT‐qPCR. RT‐qPCR was performed and analyzed using the QuantStudio 3 Real‐Time PCR system (Thermo Fisher Scientific). Primer sequences are listed in Table S2.

### 2.10 Immunofluorescence Staining

Immunofluorescence staining was performed as described previously [40]. Fixed cells or lung sections were incubated with monoclonal anti‐CD59 antibody, polyclonal anti‐C5b‐9 antibody, monoclonal anti‐Annexin V antibody, polyclonal anti‐complement C9 antibody, monoclonal anti‐cleaved Caspase‐3 antibody, or anti‐CD31 antibody overnight at 4°C. Then the cells or lung sections were washed with PBS and incubated with IgG‐Alexa Fluor 488 or 594 (Thermo Fisher Scientific). The DAPI‐containing mounting medium (ab104139, Abcam) was used for mounting and staining the nuclei. Imaging was conducted using a Carl Zeiss Observer Z1 microscope, and the fluorescence intensity was quantified using ImageJ software.

### 2.11 Single‐Cell Bioinformatic Analysis of Human *CD59* and Mouse *Cd59* Expression

*CD59* gene expression data at single‐cell resolution in the human lung were obtained from the Human Protein Atlas database (accessed on 11/05/2025). GraphPad Prism software (Boston, MA, USA) was used to graphically represent the normalized transcripts per million (nTPM) of *CD59* across individual lung cell types.

Single‐cell RNA‐sequencing datasets GSE148499 and GSE207651 were retrieved from GEO (accessed on 3/30/2026). Raw gene expression matrices were processed using the Seurat package in R. After quality‐control filtering to remove cells with low feature counts or high mitochondrial gene content, data were log‐normalized and subjected to principal component analysis (PCA) and UMAP dimensionality reduction. In GSE148499, lung endothelial cells from control and LPS‐treated mice (6 and 24 h) were analyzed [41]. In GSE207651, lung cells from sham, CLP, and CLP‐24 h mice were analyzed [42]. Gene identifiers were mapped to gene symbols, and *Cd59* expression was extracted and compared across cell populations and experimental conditions.

### 2.12 Lactate Dehydrogenase Assay

Cytotoxicity was quantified using the Lactate Dehydrogenase (LDH) Assay Kit, following the manufacturer's protocol (MedChemExpress). Briefly, HULEC‐5a cells were seeded in a 96‐well plate in 100 µL medium and treated for 24 h as mentioned before. Maximum LDH release wells were generated by adding 10 µL of lysis solution, whereas spontaneous LDH release wells received 10 µL of medium and were incubated for 30 min. For LDH detection, after which 50 µL of working solution was added to each well and incubated at room temperature for 30 min, protected from light. The reaction was terminated by adding 50 µL Stop Solution, and absorbance was measured at 490 nm using a microplate reader to determine LDH activity.

### 2.13 Statistical Analysis

Data in this study were presented as means ± SD unless otherwise indicated. Statistical analyses were performed using SigmaPlot 13.0 (Systat Software). Normality was assessed before statistical testing. Comparisons between two independent groups were performed using a two‐tailed unpaired Student's *t*‐test for normally distributed data or the Mann‐Whitney U test for non‐normally distributed data. Comparisons among three or more independent groups were performed using one‐way ANOVA followed by Tukey's multiple‐comparisons test for normally distributed data or the Kruskal–Wallis test followed by Dunn's multiple‐comparisons test for non‐normally distributed data. Survival curves were compared using the Gehan–Breslow–Wilcoxon test. *p* < 0.05 was considered statistically significant.

## 3 Results

### 3.1 CD59 is Downregulated in Murine Lungs During ALI

In C57BL/6J wild‐type (WT) mice subjected to *K. p*‐induced ALI, *Cd59* mRNA expression level in lung tissues was significantly reduced (Figure 1A). Consistently, CD59 protein level also declined following *K. p* infection (Figure 1B–D). Our analyses further showed that soluble CD59 protein levels in BALF were significantly decreased after infection (Figure 1E). To determine whether CD59 downregulation is conserved across distinct ALI etiologies, we next examined *Cd59* expression in an H1N1‑induced ALI model. In mice infected with H1N1 influenza virus, *Cd59* mRNA expression in lung tissues progressively decreased over days 1, 3, and 7 p.i., demonstrating a time‐dependent decrease of CD59 during H1N1‑induced lung injury.

**FIGURE 1:**
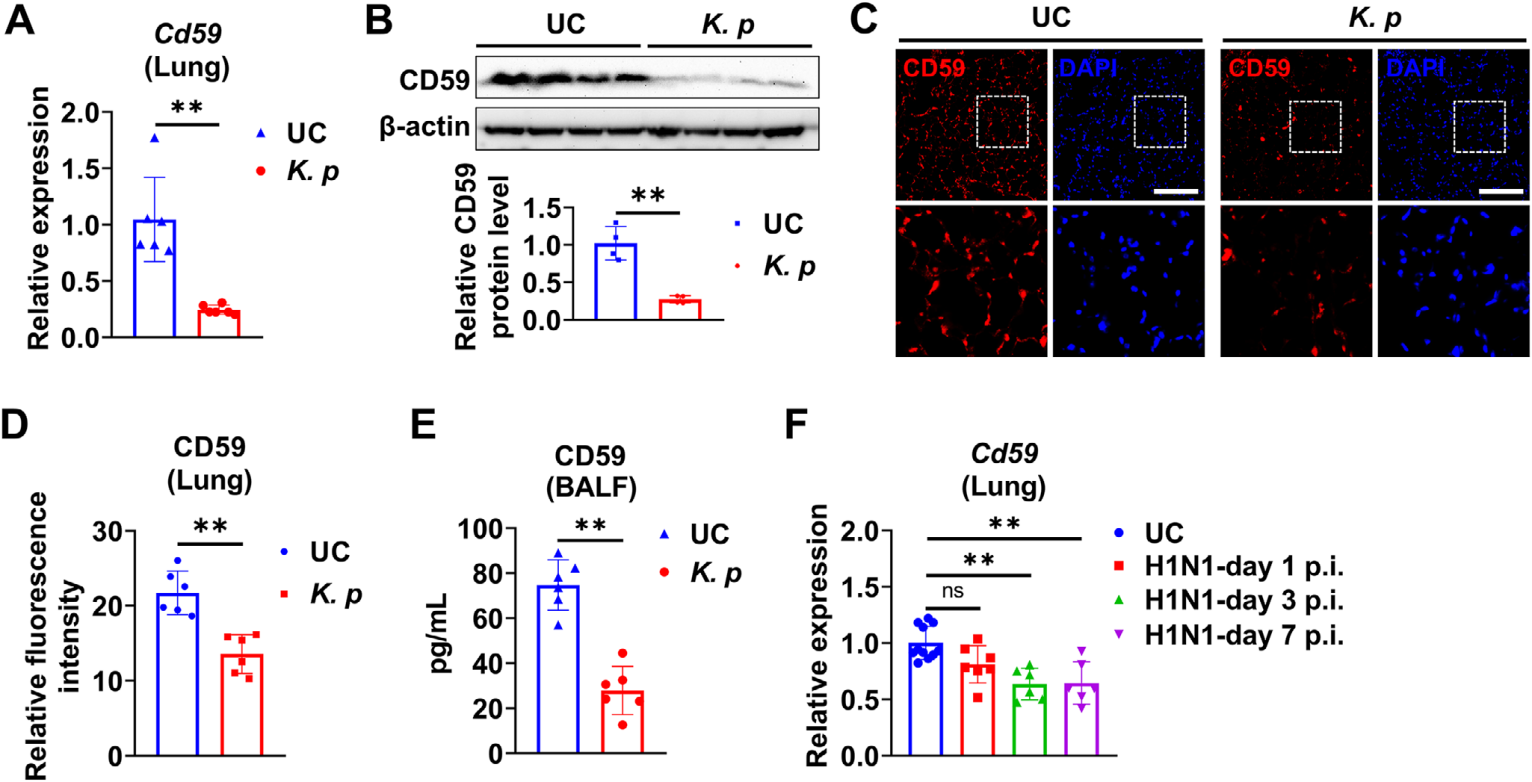
CD59 is downregulated in murine lungs during ALI. (A–E) C57BL/6J WT mice (*n* = 4–6 per group) received 50 µL PBS (Uninfected control, UC) or 50 µL PBS containing 10^4^ CFU *K. p* via i.t. Lung tissues and BALF were collected at 24 h p.i. (A) *Cd59* mRNA level in lungs was measured by RT‐qPCR. (B) CD59 protein levels in lung homogenates were quantified by Western blotting. (C) Representative immunofluorescence staining of CD59 in lung sections. (D) Quantification of CD59 immunofluorescence intensity using ImageJ. DAPI was used for nucleus staining. Scale bar = 100 µm. (E) BALF soluble CD59 protein concentrations were measured by ELISA. (F) WT mice (*n* = 6–11 per group) were infected with 10^3^ plaque‐forming units (PFU) A/California/04/2009 H1N1 in 50 µL PBS and analyzed on days 1, 3, and 7 p.i. Mice receiving PBS served as uninfected controls. *Cd59* mRNA level in murine lungs was measured by RT‑qPCR. Results represent means ± SD; ns, *p* > 0.05; ^**^, *p* < 0.01.

### 3.2 CD59 is Downregulated in ECs During ALI

We next examined *CD59* mRNA expression at single‐cell resolution in the human lung and found that *CD59* transcripts were predominantly enriched in ECs (Figure 2A). Consistent with this observation, double immunofluorescence staining further demonstrated a significant reduction in CD59 expression in lung ECs from *K. p‐*infected mice compared with uninfected controls (Figure 2B,C). In vitro, *Cd59* mRNA levels were significantly reduced in primary murine ECs following infection (Figure 2D). Similarly, CD59 protein levels were also significantly decreased in HULEC‐5a cells after infection (Figure 2E,F). To determine whether CD59 downregulation is a common feature of ALI, we further analyzed published single‐cell RNA‐sequencing datasets from LPS‐induced (GSE148499) [41] and cecal ligation and puncture (CLP; GSE207651) [42] mouse models. In both datasets, *Cd59* expression was significantly decreased in lung ECs (Figure 2G,H).

**FIGURE 2:**
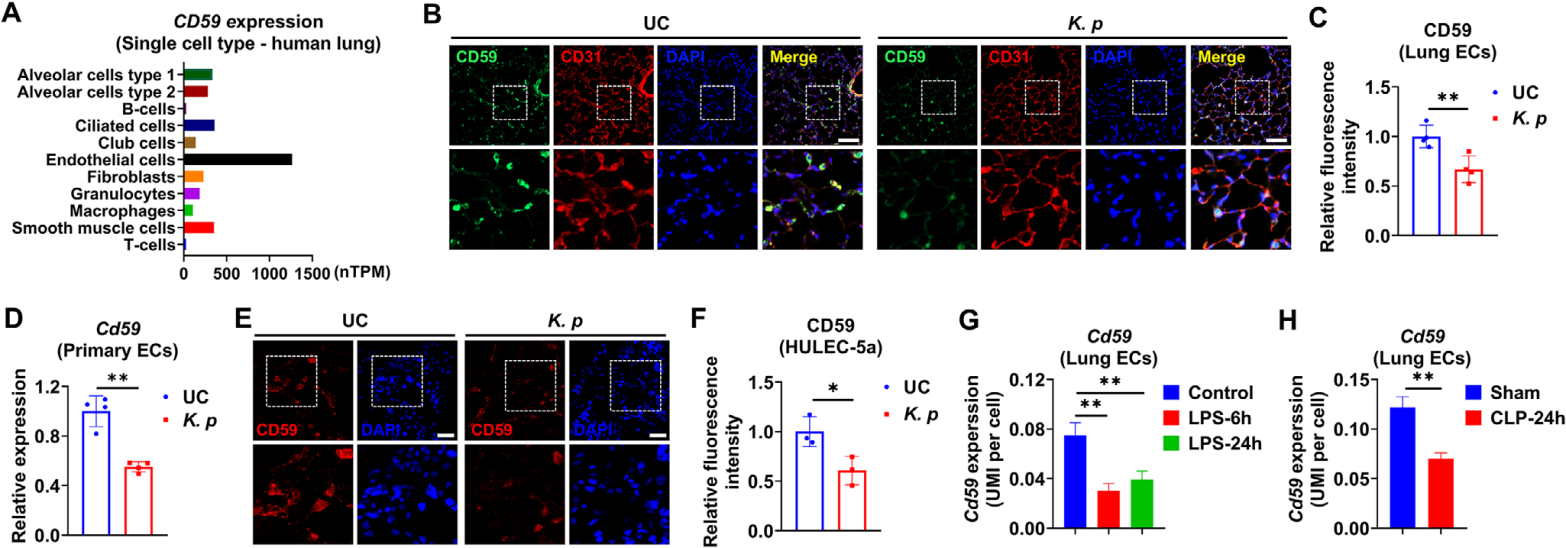
CD59 is downregulated in ECs in response to *K. p* infection. (A) Single‐cell RNA‐sequencing data from the Human Protein Atlas database show relative *CD59* mRNA expression across human lung cell populations (accessed on 11/05/2025). (B and C) WT mice (*n* = 4 per group) received 10^4^ CFU *K. p* or PBS via i.t. Lung tissues were collected 24 h p.i. (B) Double immunofluorescence staining was performed using antibodies against CD59 and the endothelial marker CD31. (C) Quantification of CD59 fluorescence intensity using ImageJ. (D) Primary murine lung ECs (*n* = 4 per group) were infected with *K. p*, and *Cd59* mRNA levels were measured 24 h p.i. by RT‑qPCR. Uninfected cells served as controls. (E and F) HULEC‐5a cells (*n* = 3 per group) were infected with *K. p*. Uninfected cells served as the control group. (E) CD59 protein expression was determined 24 h p.i. using immunofluorescence staining. (F) Fluorescence intensity was quantified using ImageJ. DAPI was used for nucleus staining. Scale bar = 50 µm. All in vitro experiments were conducted in at least three independent biological replicates. Results represent means ± SD. (G and H) Single‐cell RNA‐sequencing analysis of Cd59 expression in lung ECs from GSE148499 following LPS challenge (G) and GSE207651 comparing sham and CLP‐24 h groups (H). Data are presented as mean ± SEM. ^*^, *p* < 0.05; ^**^, *p* < 0.01.

### 3.3 *Cd59* Deficiency Exacerbates Bacteria‐Induced Lung Injury and Vascular Leakage

To define the functional role of CD59 in ALI, we utilized *Cd59* knockout (*Cd59* ^−^ *^/^* ^−^) mice and confirmed the absence of CD59 protein expression in lung tissue (Figure 3A). Following *K. p* infection, *Cd59* ^−^ *^/^* ^−^ mice exhibited increased morbidity (Figure 3B) and greater body weight loss (Figure 3C) compared with WT controls. Consistently, *Cd59* deficiency significantly exacerbated bacteria‐induced pulmonary edema and lung injury (Figure 3D–F). Moreover, Evans blue dye extravasation assays revealed substantially increased vascular leakage in *Cd59* ^−^ *^/^* ^−^ mice relative to WT mice (Figure 3G,H), indicating impaired endothelial barrier integrity.

**FIGURE 3:**
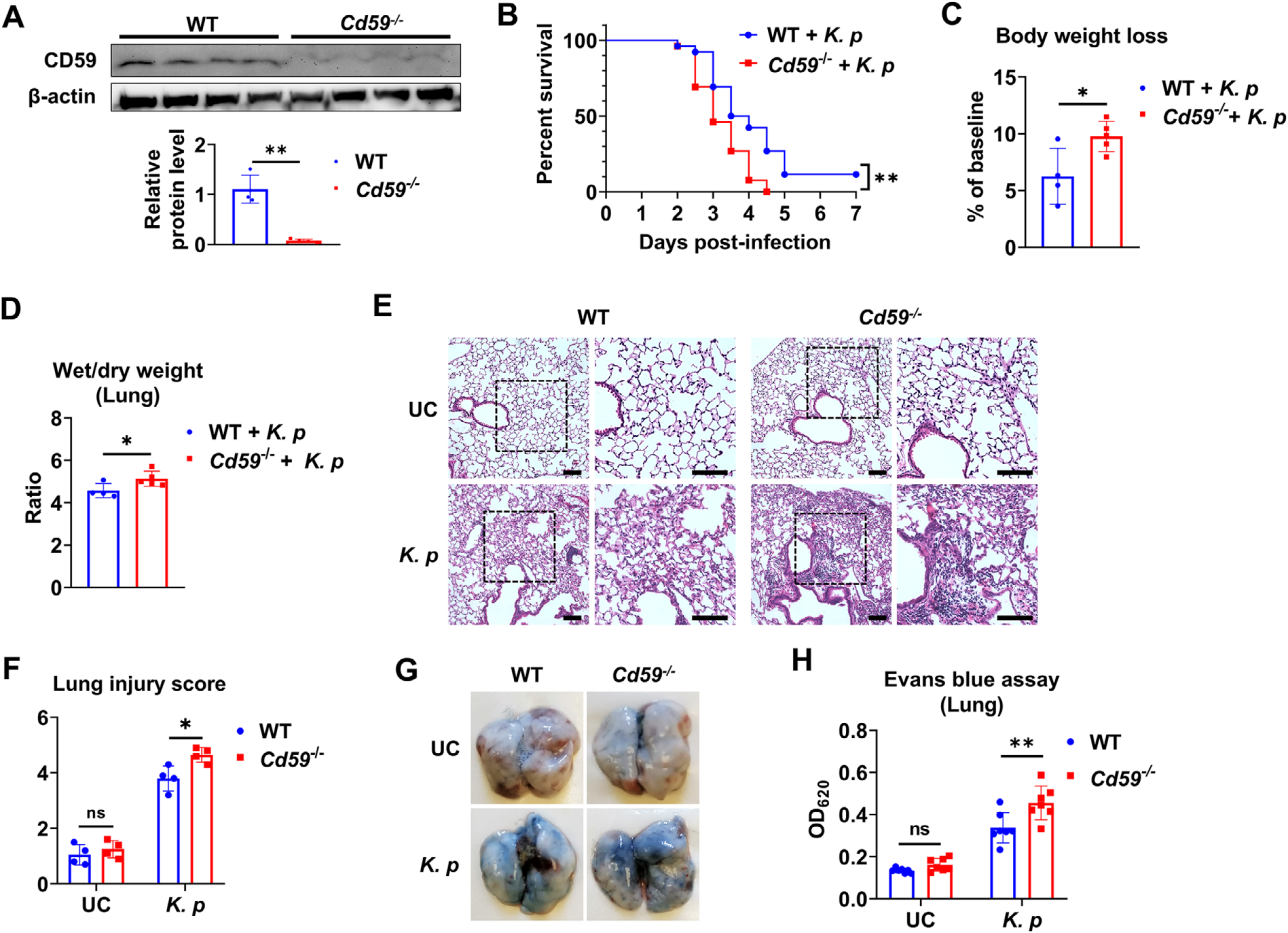
*Cd59* deficiency exacerbates bacteria‐induced lung injury and vascular leakage. (A) CD59 protein levels in lung tissues from uninfected WT and *Cd59* ^−^ *^/^* ^−^ mice were assessed by Western blotting. (B) WT and *Cd59* ^−^ *^/^* ^−^ mice (*n* = 26 per group) were infected intratracheally with 10^3^ CFU *K. p*. Survival was monitored daily, and Kaplan–Meier survival curves were generated. Differences between groups were analyzed using the Gehan‐Breslow analysis. (C–H) WT and *Cd59* ^−^ *^/^* ^−^ mice (*n* = 4–5 per group) were infected intratracheally with 10^4^ CFU *K. p* and euthanized 24 h p.i. (C) Body weight loss, (D) lung wet/dry ratio, and lung injury were compared between WT and *Cd59* ^−^ *^/^* ^−^ mice. (E) Representative H&E staining of lung sections from WT and *Cd59* ^−^ *^/^* ^−^ mice. Scale bar = 100 µm. (F) Lung injury scores were quantified and compared between groups. (G) Vascular permeability was assessed by Evans blue dye extravasation. (H) Extracted dye was quantified by measuring absorbance at 620 nm. Results represent means ± SD; ns, *p* > 0.05; ^*^, *p* < 0.05, ^**^, *p* < 0.01.

### 3.4 Loss of *Cd59* Increases MAC Deposition and Apoptosis in Lung ECs During ALI

In *Cd59* ^−^ *^/^* ^−^ mice subjected to bacteria‑induced ALI, we observed a significant increase in MAC deposition on lung ECs compared with WT controls, indicating increased susceptibility to complement‑mediated injury (Figure 4A,B). This exaggerated MAC accumulation was accompanied by significantly elevated cleaved Caspase‑3 levels (Figure 4C,D), indicative of increased endothelial apoptosis. To further establish the direct protective effect of CD59 against complement‐mediated endothelial injury, primary murine lung ECs were exposed to normal human serum (NHS) or heat‐inactivated human serum (HIHS), with or without recombinant CD59 (rCD59). NHS induced robust C9 deposition, which was significantly attenuated by rCD59 treatment (Figure S1A,B). Consistently, rCD59 decreased NHS‐induced cytotoxicity, as assessed by LDH release (Figure S1C). Together, these loss‐of‐function and rescue experiments demonstrate that CD59 protects lung ECs by limiting complement‐dependent MAC deposition and endothelial cell death.

**FIGURE 4:**
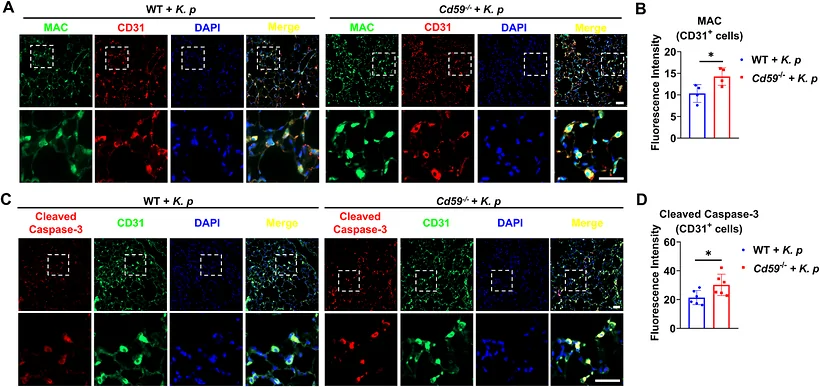
*Cd59* deficiency amplifies MAC deposition and endothelial cell death. WT and *Cd59* ^−^ *^/^* ^−^ mice (*n* = 4–6 per group) were infected intratracheally with 10^4^ CFU *K. p*. Lung tissues were collected 24 h p.i. (A and B) Lung sections were subjected to double immunofluorescence staining using antibodies against MAC and CD31. (A) Representative images of MAC staining in CD31^+^ cells. (B) MAC fluorescence intensity within CD31‑positive regions was quantified using ImageJ. (C and D) Cleaved Caspase‑3 staining was performed to assess endothelial apoptosis. (C) Representative images showing cleaved Caspase‑3 in CD31^+^ cells. (D) Fluorescence intensity was quantified using ImageJ. Scale bar = 50 µm. Results represent means ± SD; ^*^, *p* < 0.05.

### 3.5 LFA‐1 Engineering Enhances EV Localization to ECs

To generate EV^LFA−1^, we transfected HEK293T cells with plasmids encoding the αL (CD11a) and β2 (CD18) integrin subunits (Figure 5A). EVs isolated from vector‑transfected cells served as controls (EV^Con^). Western blotting confirmed the expression of canonical EV markers and the incorporation of CD11a and CD18 into EV^LFA−1^ (Figure 5B). We next examined the biodistribution of intravenously administered DiR‐labeled EVs in LPS‐treated mice. Both EV^Con^ and EV^LFA−1^ accumulated predominantly in the liver, spleen, and lung (Figure S2A,B). In lung tissue, EV^Con^ signals were primarily localized to CD68‑positive macrophages and showed minimal colocalization with CD31‑positive ECs (Figure 5C). In contrast, EV^LFA−1^ displayed greater colocalization with CD31‑positive ECs in the lungs (Figure 5D), as well as in the spleen and liver (Figure S2C,D). These findings indicate that LFA‐1 engineering enhances EV localization to vascular ECs.

**FIGURE 5:**
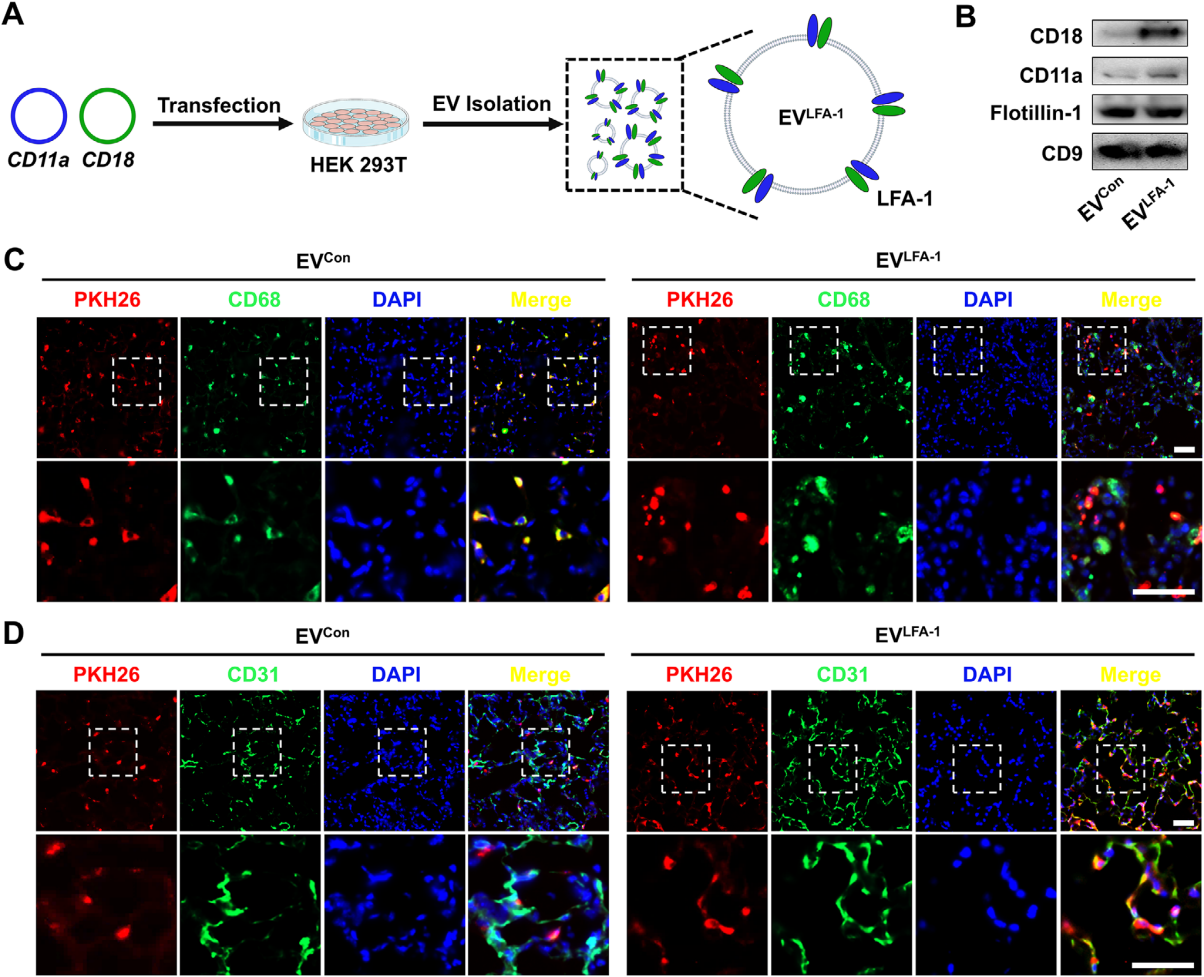
LFA‐1 engineering enhances EV localization to ECs. (A) Schematic illustration of the generation of LFA‐1‐engineered EVs. HEK293T cells were transfected with plasmids encoding CD11a and CD18, or vector control plasmids. EV^Con^ and EV^LFA‑1^ were subsequently isolated from conditioned media by ultracentrifugation. (B) Western blot analysis using 50 µg EV protein lysates, showing expression of CD11a, CD18, and EV markers. (C and D) WT mice (*n* = 4 per group) were treated intratracheally with LPS (5 µg per mouse). After 24 h, mice received an intravenous injection of 200 µg PKH26‑labeled EV^Con^ or EV^LFA‑1^ and were euthanized 24 h later. Lung sections were stained with antibodies against CD68 (C) or CD31 (D). Nuclei were counterstained with DAPI. Scale bar = 50 µm. All in vitro experiments were conducted in three independent biological replicates.

### 3.6 LFA‐1 Engineering Enhances EV‐Mediated Delivery of CD59 to ECs

To restore endothelial CD59 levels, we generated CD59‐loaded, LFA‐1‐engineered EVs (EV^LFA−1^‐CD59) by transfecting HEK293T cells with plasmids encoding CD59 and the LFA‐1 subunits CD11a and CD18 (Figure 6A). Western blotting confirmed the incorporation of CD11a, CD18, and CD59 into EV^LFA−1^‐CD59, together with the canonical EV markers flotillin‐1, SP‐1, and CD9 (Figure 6B). Transmission electron microscopy (TEM) coupled with immunogold labeling further demonstrated CD59 localization on the EV surface (Figure 6C), while nanoparticle tracking analysis (NTA) showed comparable size distributions between EV^LFA−1^ and EV^LFA−1^‐CD59 (Figure 6D). We next evaluated CD59 delivery to *Cd59* ^−/−^ primary ECs. CD59‐loaded EVs lacking LFA‐1 (EV‐CD59) increased cell‐associated CD59 levels compared with EV^Con^, whereas EV^LFA−1^‐CD59 resulted in a further increase in cell‐associated CD59 accumulation (Figure 6E,F). These findings demonstrate that LFA‐1 engineering enhances EV‐mediated CD59 delivery to ECs.

**FIGURE 6:**
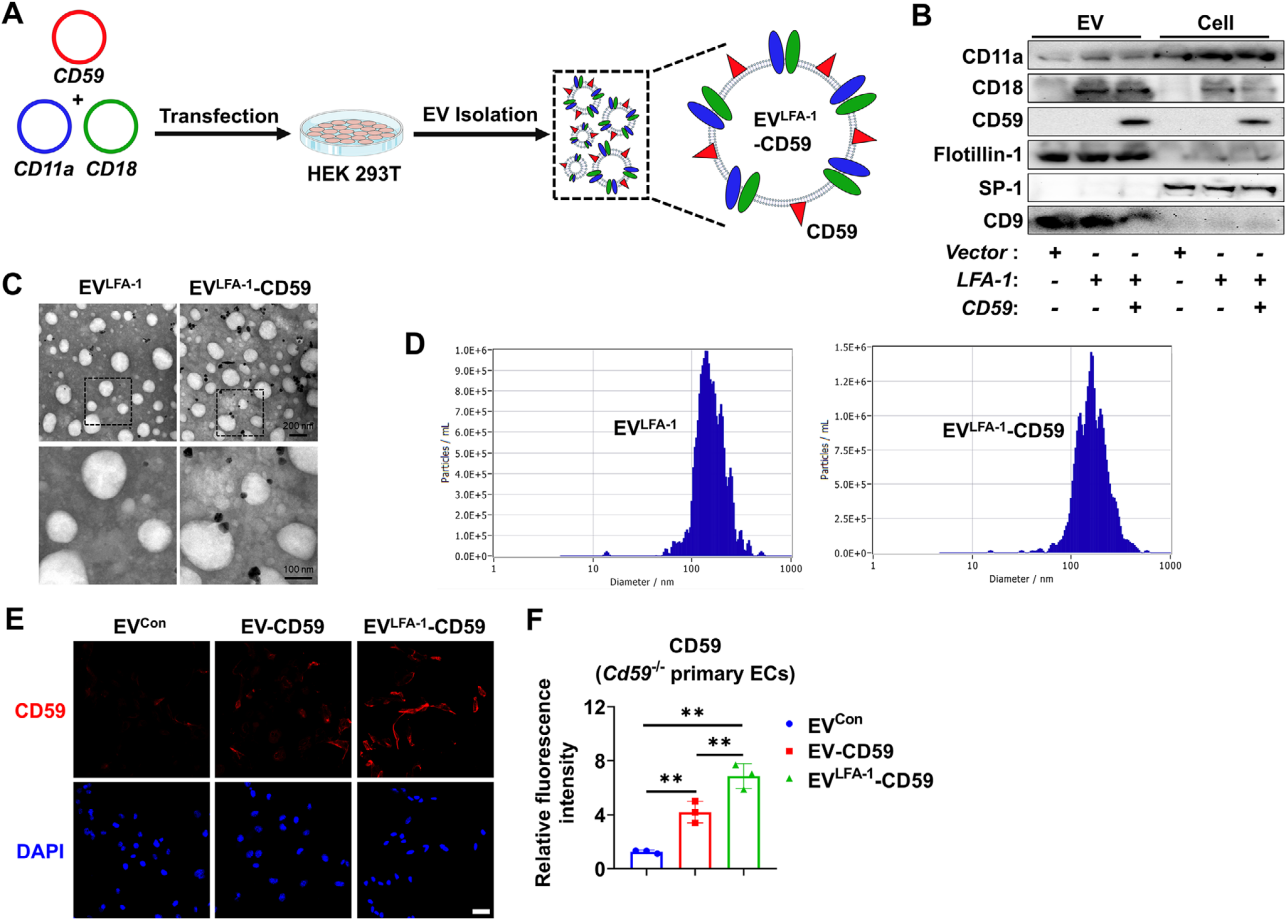
Engineered EV^LFA−1^ enriched with CD59 improves survival following *K. p* infection. (A) Schematic illustration of the generation of EV^LFA−1^‐CD59. (B) Western blot analysis using 50 µg EV or corresponding cell lysates following transfection, showing expression of CD11a, CD18, CD59, SP‐1, and EV markers Flotillin‐1 and CD9. (C) Representative TEM images of EV^LFA−1^ and EV^LFA−1^‐CD59 are shown. EVs labeled with anti‑CD59 immunogold staining were visualized by transmission electron microscopy. (D) The size distribution of EV^LFA−1^ and EV^LFA−1^‐CD59 was determined by NTA. All in vitro experiments were conducted in three independent biological replicates. (E and F) Primary lung ECs isolated from *Cd59^−^^/^^−^* mice (*n* = 4) were incubated with EV‐CD59 or EV^LFA−1^‐CD59 (50 µg/mL) for 2 h in the presence of heat‐inactivated normal human serum (HIHS). Representative CD59 immunofluorescence images showing cell‐associated CD59 (E). Quantification of CD59 fluorescence intensity using ImageJ (F). Nuclei were counterstained with DAPI. Scale bar = 50 µm. ^**^, *p* < 0.01.

### 3.7 EV^LFA−1^‐CD59 Protects ECs from Complement‐Mediated Injury and Improves Survival Following *K. p* Infection

We next assessed whether EV‐carried CD59 retained its complement‐inhibitory activity. In HULEC‐5a cells exposed to NHS, EV^LFA−1^‐CD59 significantly reduced MAC deposition and LDH release compared with EV^LFA−1^ (Figure 7A–C). Similarly, EV^LFA−1^‐CD59 reduced MAC deposition and Annexin V staining in *Cd59* ^−/−^ primary ECs (Figure 7D–F), demonstrating protection against complement‐mediated endothelial injury and apoptosis. To evaluate their therapeutic potential, mice were treated with EV^LFA−1^ or EV^LFA−1^‐CD59 at 24 h after *K. p* infection (Figure 7G). Notably, EV^LFA−1^‐CD59 treatment significantly improved survival compared with EV^LFA−1^ alone (Figure 7H). However, BALF C5a and C9 levels were comparable between the two groups (Figure S3A,B), indicating that its protective effect was not associated with broad suppression or depletion of pulmonary complement components.

**FIGURE 7:**
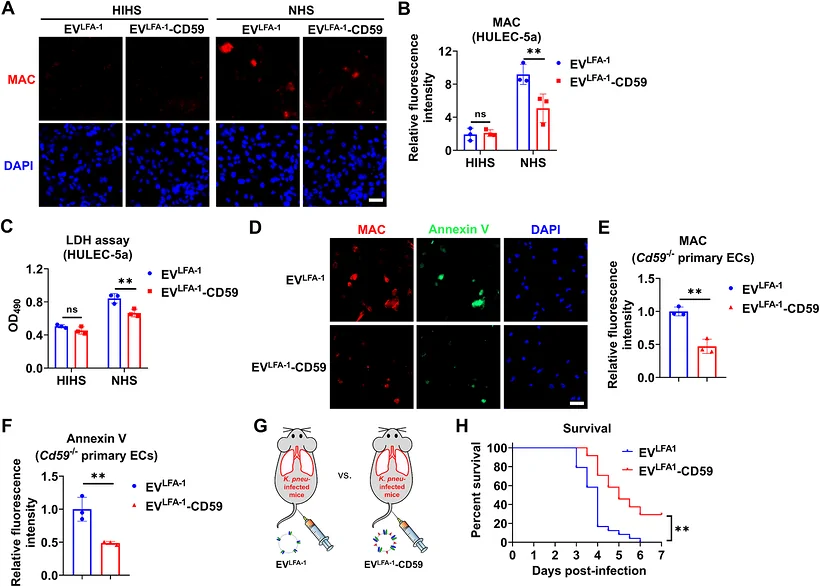
EV^LFA−1^‐CD59 protects ECs from complement‐mediated injury and improves survival following *K. p* infection. (A–C) HULEC‐5a cells were treated with 50 µg/mL EV^LFA−1^ or EV^LFA−1^‐CD59 in the presence of heat‐inactivated normal human serum (HIHS) or normal human serum (NHS). (A) Immunofluorescence staining was performed with antibodies against MAC. Nuclei were counterstained with DAPI. Scale bar = 50 µm. Fluorescence intensity was quantified using ImageJ (B). Complement‑dependent cytotoxicity was determined by LDH activity in the culture medium (C). (D–F) Primary lung ECs were isolated from *Cd59^−^/^−^* mice (*n* = 3) incubated with normal human serum (NHS), with 50 µg/mL EV^LFA−1^ or EV^LFA−1^‐CD59. Representative fluorescence images showing MAC deposition detected by immunostaining and apoptosis assessed by Annexin V staining (D). Quantification of MAC (E) and Annexin V (F) fluorescence intensities using ImageJ. Nuclei were counterstained with DAPI. Scale bar = 50 µm. (G) Schematic illustration of animal study design. (H) WT mice (*n* = 24 per group) were intratracheally infected with 10^3^ CFU *K. p*. At 24 h p.i., mice received 200 µg EV^LFA−1^ or EV^LFA−1^‐CD59 via intravenous injection. Survival was monitored daily from the time of infection, and Kaplan–Meier survival curves were generated. Differences between groups were analyzed using the Gehan–Breslow analysis. ^**^, *p* < 0.01.

### 3.8 Intravenous EV^LFA−1^‐CD59 Delivery Protects Mice From Lung Vascular Leakage During ALI

Having established that EV^LFA−1^‐CD59 improved survival following *K. p* infection (Figure 7H), we next included two additional control groups, EV^Con^ and EV‐CD59, to distinguish the contributions of CD59 loading and LFA‐1‐mediated targeting. EV^LFA−1^‐CD59 significantly reduced pulmonary Evans blue extravasation, whereas EV‐CD59 and EV^LFA−1^ did not significantly reduce it over EV^Con^ (Figure 8A,B). A similar pattern was observed for lung edema and alveolar hemorrhage, as assessed by the lung wet‐to‐dry weight ratio and BALF RBC counts, respectively (Figure 8C,D). Histological analysis further showed that EV^LFA−1^‐CD59 preserved alveolar architecture and reduced lung injury, while neither EV‐CD59 nor EV^LFA−1^ alone conferred significant protection (Figure 8E,F). EV^LFA−1^‐CD59 reduced BALF IL‐1β and TNF‐α levels compared with EV^Con^, whereas EV‐CD59 and EV^LFA−1^ alone did not significantly affect either cytokine (Figure S4A,B). Together, these findings demonstrate that CD59 loading alone is insufficient and support a critical role for LFA‐1‐mediated targeting in the protective efficacy of EV‐delivered CD59 during *K. p* infection.

**FIGURE 8:**
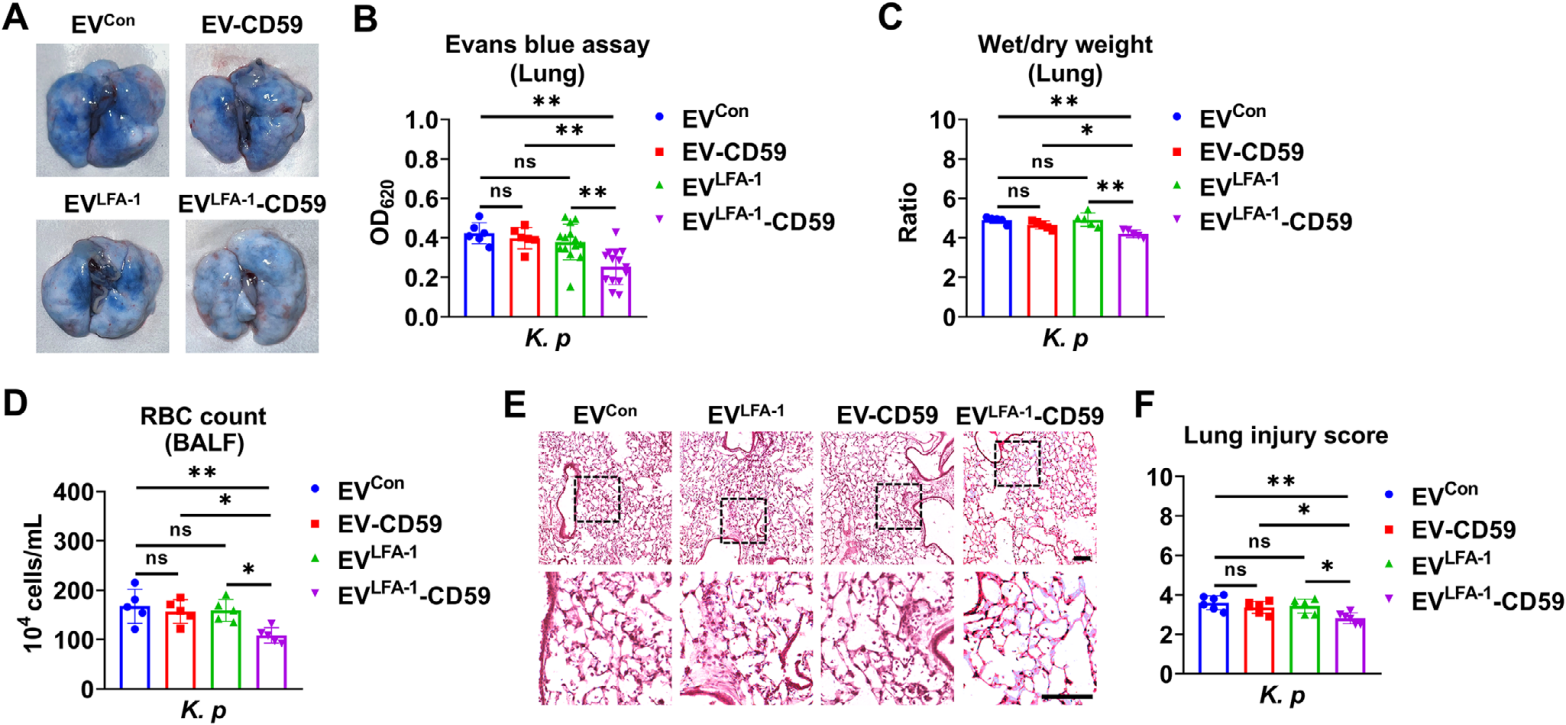
Intravenous EV^LFA−1^‐CD59 delivery protects mice from lung vascular leakage during ALI. WT mice were intratracheally infected with 10^4^ CFU *K. p*. At 24 h p.i., mice received 200 µg of EV^Con^, EV‐CD59, EV^LFA−1,^ or EV^LFA−1^‐CD59 intravenously and were euthanized 24 h after EV administration. (A and B) Representative images and quantification of pulmonary Evans blue extravasation (*n* = 6–14 per group). (C) Lung wet‐to‐dry weight ratios (*n* = 5 per group). (D) RBC counts in BALF (*n* = 5 per group). (E and F) Representative H&E‐stained lung sections and quantification of lung injury scores (*n* = 6 per group). Scale bar = 100 µm. Data are presented as mean ± SD. ns, *p* > 0.05; ^*^, *p* < 0.05; ^**^, *p* < 0.01.

### 3.9 Intravenous EV^LFA−1^‐CD59 Delivery Limits MAC Deposition and Apoptosis in Lung ECs

To investigate the mechanism underlying the protection conferred by EV^LFA−1^‐CD59, we assessed MAC deposition and apoptosis in lung EVs after *K. p* infection. Immunofluorescence staining revealed that EV^LFA−1^‐CD59 significantly reduced MAC deposition (Figure 9A,B) and cleaved Caspase‐3 (Figure 9C,D) staining in CD31‐positive lung cells, whereas EV‐CD59 and EV^LFA−1^ showed no significant effects compared with EV^Con^. Together, these findings show that LFA‐1‐targeted delivery enables EV‐associated CD59 to reduce endothelial MAC deposition and apoptosis during *K. p* infection.

**FIGURE 9:**
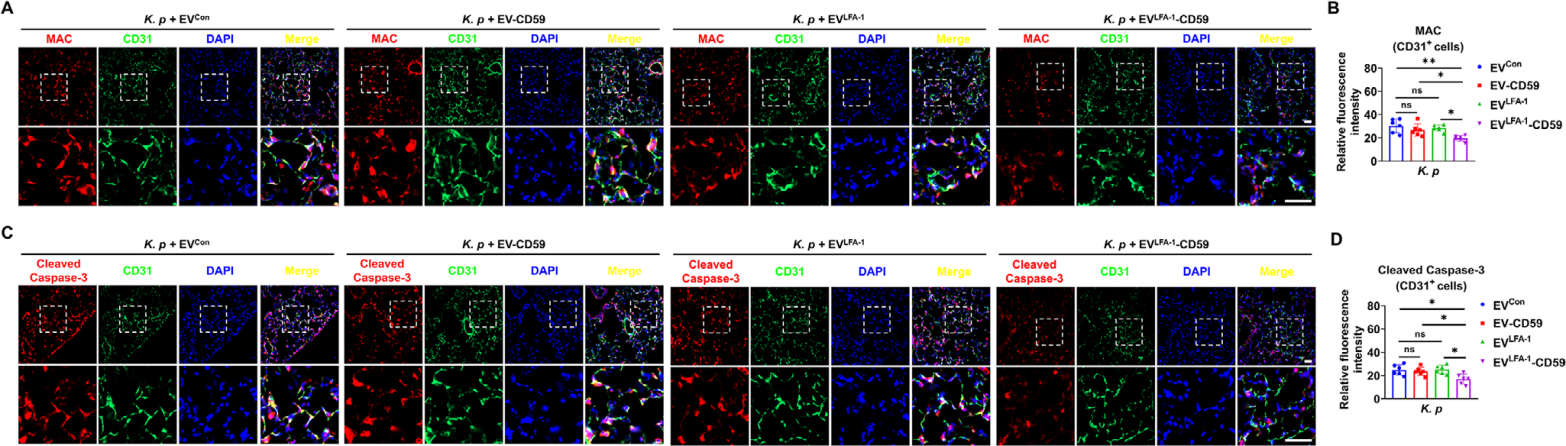
EV^LFA−1^‐CD59 limits MAC deposition and prevents endothelial cell death. WT mice were intratracheally infected with 10^4^ CFU *K. p*. At 24 h p.i., mice received 200 µg of EV^Con^, EV‐CD59, EV^LFA−1,^ or EV^LFA−1^‐CD59 intravenously and were euthanized 24 h after EV administration. Double immunofluorescence staining was performed to detect MAC deposition and apoptosis in murine lung sections (*n* = 6 per group). (A) Representative images showing MAC deposition in CD31‑positive endothelial cells. (B) Quantification of MAC fluorescence intensity in CD31‑positive cells. (C) Representative images showing cleaved Caspase‑3 in CD31‑positive endothelial cells. (D) Quantification of cleaved Caspase‑3 fluorescence intensity in CD31‑positive cells. Data are presented as mean ± SD. ns, *p* > 0.05; ^*^, *p* < 0.05; ^**^, *p* < 0.01.

## 4 Discussion

ALI remains a major cause of morbidity and mortality among critically ill patients [43]. Despite its substantial clinical impact, no therapies that reverse ALI, and pharmacologic approaches to directly correct endothelial barrier dysfunction are lacking. ALI initiates immune activation, vascular permeability, and edema, rapidly impairing gas exchange and progressing to respiratory failure [44]. Current management relies largely on supportive measures such as mechanical ventilation, which do not address the molecular drivers of endothelial injury. Defining the signaling pathways that govern endothelial permeability and dysfunction in ALI is therefore essential for developing targeted, mechanism‐based therapies that preserve lung function and improve patient outcomes.

In patients with community‐acquired pneumonia (CAP), serum C3a was significantly elevated compared with healthy controls and increased with clinical severity [45]. Elevated C3a levels are positively associated with increased inflammatory cytokines during CAP, indicating a role for ‘complement‐mediated pulmonary inflammation’. The complement system is a central component of innate immunity, functioning to eliminate pathogens and coordinate inflammatory responses [46, 47]. However, excessive or dysregulated complement activation becomes deleterious, particularly in lung disease. In ALI, increased complement activation products, including C5a and MAC, promote endothelial dysfunction, leukocyte recruitment, and increased vascular hyperpermeability, thereby exacerbating tissue injury and pulmonary edema [48, 49, 50, 51]. Clinical and experimental studies consistently demonstrate that complement hyperactivation correlates with disease severity and adverse outcomes in ALI/ARDS, underscoring its dual protective and injurious roles [48, 50, 52].

CD59 is the principal membrane‐bound inhibitor of MAC, and sustained complement activation may overwhelm or destabilize CD59‐mediated regulation at the alveolar‐capillary interface, thereby contributing to progressive lung injury. Mechanistically, CD59 acts selectively at the terminal stage of the complement cascade by binding C8 and C9 within nascent MAC intermediates [53, 54], thereby preventing their membrane insertion and the subsequent C9 polymerization required for formation of the lytic pore [55]. Consistent with this mechanism, recombinant CD59 reduced serum‐induced C9 deposition and cytotoxicity in primary ECs. In our study, CD59 was significantly reduced in the murine lungs during *K. p* and H1N1 influenza infection compared with uninfected controls, suggesting that *Cd59* loss may link complement dysregulation to endothelial injury in ALI. Pulmonary endothelial injury is now recognized as a central hallmark of ALI/ARDS [56]. Disruption of the lung microvascular endothelial barrier, characterized by cytoskeletal remodeling, tight junction dysregulation, endothelial inflammation, and endothelial cell death, is a key feature of both experimental and clinical ALI/ARDS [57]. Consistent with this role, *CD59* mRNA is significantly enriched in ECs relative to other lung cell populations. Both CD59 mRNA and protein levels were significantly reduced in lung ECs during *K. p* infection. Downregulation of CD59 has also been reported in Shiga toxin 2‐induced injury in human renal tubular epithelial cells and glomerular ECs [58]. Collectively, these findings support the concept that CD59 serves as a critical regulator of complement activity at the alveolar‐capillary level and identify CD59 as a promising molecular target for mitigating complement‐mediated endothelial injury in ALI.

Beyond its pore‐forming activity, MAC (C5b‐9) also functions as a potent inflammatory stimulus in endothelial and other nucleated cells. Sublytic MAC deposition on human ECs induces IL‐8 and monocyte chemoattractant protein‐1 expression through NF‐κB activation, directly linking complement activation to inflammatory signaling [59]. Additional mechanistic studies demonstrate that MAC triggers cellular stress and NLRP3 inflammasome activation, leading to the release of pro‐inflammatory cytokines such as IL‐1β and IL‐18, and thereby amplifying tissue injury, including renal tubular epithelial cell damage [60]. CD59 functions not only as a structural inhibitor of MAC assembly but also as an endogenous anti‑inflammatory mediator. Loss of CD59 function during viral or bacterial challenge may amplify complement‐driven endothelial activation and tissue injury. In this study, WT mice exhibited better survival than *Cd59* ^−^ *^/^* ^−^ mice, with less weight loss, inflammatory infiltration, and lung injury following *K. p* infection, indicating that loss of *Cd59* enhances complement‑driven inflammatory signaling. Consistent with these findings, Longhi and colleagues reported increased T cell and neutrophil recruitment in *Cd59*‐deficient mice and worsened influenza‐induced pulmonary inflammation [61]. Moreover, the Evans blue assay revealed significantly greater vascular leakage in *Cd59* ^−^ *^/^* ^−^ mice than in WT controls. This phenotype was associated with increased cleaved Caspase‐3 levels in CD31‐positive (CD31^+^) ECs during *K. p* infection, indicating that reduced CD59 may contribute to dysregulated complement activation, endothelial apoptosis, and vascular barrier failure. Supporting a broader anti‐apoptotic role for CD59, prior studies have shown that CD59 overexpression suppresses apoptosis in T‐acute lymphoblastic leukemia via the AKT pathway [62]. Collectively, these findings indicate that strategies designed to preserve or restore CD59‐mediated regulation may represent a promising therapeutic approach to attenuate complement‐driven lung injury and maintain microvascular integrity in ALI/ARDS.

CD59 inhibits MAC assembly at the terminal step of the complement cascade, thereby preventing MAC‐mediated cell lysis [53]. However, the therapeutic efficacy of recombinant CD59 was limited when administered alone [63]. Compared with conventional delivery methods, EVs offer distinct advantages derived from their cellular origin, including enhanced biocompatibility and low immunogenicity. The native lipid bilayer of EVs facilitates membrane fusion and efficient cytoplasmic delivery of cargo following endosomal uptake, enhancing functional payload transfer to recipient cells [20, 64]. Despite these advantages, intrapulmonary administration of EVs results in rapid uptake by alveolar macrophages, which represent a major clearance pathway within the alveolar space [65]. Although cell‑specific EV targeting, including damaged lung ECs during ALI, is achievable, efficacy in bacteria‐induced vascular permeability remains limited [65, 66]. To overcome this barrier, we engineered EVs to express LFA‐1 (EV^LFA−1^), enabling binding to ICAM‑1, which is robustly upregulated on activated pulmonary ECs during ALI. This strategy provides a rational approach for targeting injured microvascular endothelium [27, 67]. Targeted LFA‐1 expression enables the enrichment of CD59 at the endothelial surface, where terminal complement activation occurs, potentially providing more effective control of MAC assembly than untargeted recombinant protein therapy. Moreover, EV‐based delivery maintains CD59 within the lipid bilayer microenvironment, preserving its physiological orientation and functional activity. Dual‐immunofluorescence analysis indicated that PKH‐26‐labeled EV^LFA−1^ was preferentially co‐localized with CD31‐positive cells compared with control EVs. We further engineered CD59 into EV^LFA−1,^ generating EV^LFA−1^‐CD59. Notably, mice administered EV^LFA−1^‐CD59 exhibited significantly reduced *K. p*‐induced lung injury and vascular leakage compared with mice receiving EV^LFA−1^. To our knowledge, this is the first demonstration of LFA‑1‐directed CD59 delivery to lung ECs conferring protection during ALI. Consistent with the role of CD59 as the principal inhibitor of MAC assembly, treatment with EV^LFA−1^‐CD59 significantly reduced complement deposition in the injured lung, as evidenced by a decrease in MAC formation on lung ECs. Notably, EV^LFA−1^‐CD59 treatment also suppressed cleaved Caspase‐3 levels, indicating that targeted delivery of CD59 not only prevents membrane‐disruptive injury but also reduces apoptotic signaling in lung ECs. Together, these findings support a model in which targeted delivery of CD59 to ICAM‐1‐activated endothelium stabilizes vascular integrity by limiting complement‐driven cell injury and preventing progression to apoptosis. EV^LFA−1^‐CD59, therefore, represents a promising strategy to restore endogenous complement regulation at sites of vascular injury and to mitigate endothelial damage during ALI.

Several limitations of the current study warrant consideration and provide potential directions for future investigation. First, our in vivo studies used global *Cd59*‐deficient mice, precluding definitive attribution of observed effects to endothelial cell‐specific CD59 loss. Future studies using endothelial‐specific *Cd59* conditional knockout models, such as VE‐cadherin Cre‐mediated deletion, will be necessary to precisely define the role of endothelial CD59 in regulating complement‐mediated vascular injury during ALI. Second, although EV^LFA−1^‐CD59 showed protective effects in murine ALI, additional studies are needed to evaluate long‐term efficacy, durability, and safety. Notably, EV^LFA−1^‐CD59 reduced endothelial MAC deposition without altering BALF C5a or C9 levels, supporting a localized effect on terminal MAC assembly rather than broad inhibition of complement activation. This targeted mechanism may offer a safety advantage by limiting complement‐mediated endothelial injury while preserving the physiological roles of upstream complement pathways. However, BALF measurements alone do not fully establish systemic complement safety, and future studies should assess circulating complement activity, susceptibility to infection, immunogenicity, and repeated‐dose toxicity. In addition to inhibiting MAC formation, CD59 has complement‐independent functions, including regulation of NK, B, and T cell activities [68]; these immune‐regulatory roles were not investigated here. Finally, engineered EVs were generated using electroporation as a proof‐of‐concept approach. While effective for initial studies, this transient approach limits translational scalability and reproducibility. Future work will require the development of more stable, reproducible, and scalable strategies for producing EV^LFA−1^‐CD59 suitable for clinical or large‐scale applications.

In summary, our study establishes CD59 as a critical regulator of complement‐mediated endothelial injury in bacteria‐induced ALI. Using LFA‐1‐engineered EVs, we achieved CD59 preferential delivery to lung ECs, preserving vascular integrity and mitigating lung injury. By limiting complement‑mediated injury and endothelial apoptosis, EV^LFA−1^‐CD59 represents a targeted delivery strategy that addresses a central pathogenic mechanism in ALI/ARDS and holds promise as a novel therapeutic approach.

## Author Contributions

**Y. Z**.: Investigation, Methodology, Formal analysis, Data curation, Writing – review & editing, Writing – original draft. **SE**: Methodology, Investigation, Formal analysis, Data curation. **Y. L., S. P. N.,** and **P. R. S**.: Supervision, Resources, Writing – review & editing, Conceptualization. **X. W**.: Methodology, Supervision, Resources, Writing – review & editing. **D. Z**.: Supervision, Resources, Writing – review & editing, Conceptualization, Funding acquisition, Formal analysis.

## Conflicts of Interest

The authors declare no conflicts of interest.

## Supporting information




**Supporting File**: advs78673‐sup‐0001‐SuppMat.docx.

## Data Availability

The data that support the findings of this study are available from the corresponding author upon reasonable request.
