# Supplementary material for: LFA‐1‐Engineered Extracellular Vesicles Deliver CD59 to Protect Endothelial Integrity in Acute Lung Injury
Source: Adv Sci (Weinh). 2026 Nov 10:e78673. Online ahead of print. doi: 10.1002/advs.78673 (PMC13752224; doi:10.1002/advs.78673)
Supplement: Supplementary file 1 [file ADVS-9999-e78673-s001.docx]

**Supporting information**

**LFA-1-Engineered Extracellular Vesicles Deliver CD59 to Protect Endothelial Integrity in Acute Lung Injury**

Yin Zhu^1,2^, Shravani Etrouth^1^, Payaningal R. Somanath^1,2^, S Priya Narayanan^1,2^, Yutao Liu^3^, Xiaoyun Wang^1,4^, and Duo Zhang^1,2*^

^1^ Clinical and Experimental Therapeutics, Department of Clinical and Administrative Pharmacy, College of Pharmacy, University of Georgia, Augusta, GA 30912, USA

^2^ Augusta VA Healthcare System, Augusta, GA 30912, USA

^3^ Department of Ophthalmology, Mayo Clinic, Rochester, MN 55905

^4^ Pulmonary, Critical Care and Sleep Medicine, Department of Medicine, Baylor College of Medicine, Houston, Texas 77030, USA

^*^ Correspondence: Duo Zhang (duozhang@uga.edu)

**Table S1. Antibodies used in this study**

| **Antibody** | **Host species** | **Application** | **Dilution** | **Manufacturer** | **Catalog no.** |
| --- | --- | --- | --- | --- | --- |
| CD59 | Rabbit | WB/IF | WB: 1:1000  IF: 1:500 | Thermo Fisher Scientific | MA5-32588 |
| β-Actin | Mouse | WB | 1: 2000 | MilliporeSigma | A5441 |
| CD31 | Rat | IF/Primary ECs isolation | IF: 1:200 | BD Pharmingen | 553369 |
| CD102 | Rat | Primary ECs isolation | n/a | BD Pharmingen | 553325 |
| MAC | Rabbit | IF | 1:500 | Thermo Fisher Scientific | BS-2673R |
| CD11a | Rabbit | WB | 1:2000 | Abcam | ab52895 |
| CD18 | Rabbit | WB | 1:1000 | Abcam | ab307406 |
| CD9 | Rabbit | WB | 1:1000 | Abcam | ab92726 |
| Flotillin-1 | Rabbit | WB | 1:2000 | Abcam | ab133497 |
| SP-1 | Rabbit | WB | 1:500 | Abcam | ab227383 |
| CD68 | Mouse | IF | 1:250 | Abcam | ab955 |
| Cleaved Caspase-3 | Rabbit | IF | 1:750 | Cell Signaling Technology | 9664S |
| Complement C9 | Rabbit | IF | 1:500 | Thermo Fisher Scientific | PA5-29093 |
| Annexin V | Rabbit | IF | 1:100 | Abcam | ab108194 |
| HRP-conjugated anti-mouse secondary antibody | Goat | WB | 1:2000 | R&D Systems | HAF007 |
| HRP-conjugated anti-rabbit secondary antibody | Goat | WB | 1:2000 | R&D Systems | HAF008 |
| Anti-Rabbit IgG Secondary Antibody, Alexa Fluor™ 488 | Goat | IF | 1:2000 | Thermo Fisher Scientific | A-11008 |
| Anti-Rabbit IgG Secondary Antibody, Alexa Fluor™ 594 | Goat | IF | 1:2000 | Thermo Fisher Scientific | A-11012 |
| Goat anti-Rat IgG Secondary Antibody, Alexa Fluor™ 594 | Goat | IF | 1:2000 | Thermo Fisher Scientific | A-11007 |
| Anti-Rat IgG Secondary Antibody, Alexa Fluor™ 488 | Goat | IF | 1:2000 | Thermo Fisher Scientific | A-11006 |
| Goat anti-Mouse IgG Secondary Antibody, Alexa Fluor™ 488 | Goat | IF | 1:2000 | Thermo Fisher Scientific | A-11001 |

**Table S2. Primer sequences used for quantitative real-time PCR**

| **Species** | **Gene** | **Direction** | **Primer sequence (5′-3′)** |
| --- | --- | --- | --- |
| Human | *TBP* | Forward | TGTATCCACAGTGAATCTTGGTTG |
| Human | *TBP* | Reverse | GGTTCGTGGCTCTCTTATCCTC |
| Human | *CD59* | Forward | TGATGCGTGTCTCATTACCAAAGC |
| Human | *CD59* | Reverse | ACACAGGTCCTTCTTGCAGCAG |
| Mouse | *Tbp* | Forward | CTACCGTGAATCTTGGCTGTAAAC |
| Mouse | *Tbp* | Reverse | AATCAACGCAGTTGTCCGTGGC |
| Mouse | *Cd59* | Forward | CACTTGCTCTCCTGACCAGGAT |
| Mouse | *Cd59* | Reverse | CACAAGTTAAACTGGCAGCATCTG |

**Figure S1. CD59-mediated regulation of terminal complement activity and EC injury.** Primary lung ECs were isolated from *Cd59*^-/-^ mice (n = 4) were exposed to heat-inactivated normal human serum (HIHS) or normal human serum (NHS), with or without 10 μg/mL recombinant CD59 (rCD59). (A) Representative C9 immunofluorescence images. Nuclei were counterstained with DAPI. Scale bar = 50 μm. (B) Quantification of fluorescence intensity using ImageJ. (C) Cell injury assessed by lactate dehydrogenase (LDH) release. Results represent means ± SD; ns, *p* > 0.05; **, *p* < 0.01.

**Figure S2. Organ biodistribution and endothelial localization of EV^Con^ and EV^LFA-1^ in mice.** WT mice (n = 4 per group) were studied with intratracheal LPS treatment (5 μg per mouse). After 24 h, mice received an intravenous injection of DiR-labeled EV^Con^ or EV^LFA-1^ (200 μg per mouse), and were euthanized 2 h after EV administration, and the brain, heart, lungs, liver, kidneys, and spleen were collected for ex vivo fluorescence. Representative organ images (A) and fluorescence signal quantification (B) are shown. (C and D) WT mice (n = 4 per group) were studied with intratracheal LPS treatment (5 μg per mouse). 24 h later, the mice received an intravenous injection of PKH26-labeled EV^Con^ or EV^LFA-1^ (200 μg per mouse) and were euthanized 2 h after EV administration. Spleen (C) and liver (D) sections were stained with antibodies against CD31. Nuclei were counterstained with DAPI. Scale bar = 50 μm. Results represent means ± SD.

**Figure S3. Effects of EV-delivered CD59 on C5a and C9 levels after infection.** WT mice (n = 5 per group) were infected with *K. p* (10^4^ CFU) and were treated with EV^LFA-1^ or EV^LFA-1^-CD59 intravenously after 24 h. BALF concentrations of C5a (A) and C9 (B) were measured by ELISA. Results represent means ± SD; ns, *p* > 0.05.

**Figure S4. Effects of engineered EVs on pulmonary inflammatory cytokine levels following bacterial infection.** WT mice (n = 6 per group) were infected intratracheally with *K. p* (10⁴ CFU per mouse). At 24 h post-infection, mice received 200 μg of EV^Con^, EV-CD59, EV^LFA-1^ or EV^LFA-1^-CD59 intravenously and were euthanized 24 h later. (A) IL-1β and (B) TNF-α levels in BALF were quantified by ELISA. Results represent means ± SD; ns, *p* > 0.05; *, *p* < 0.05.
